# LipoxinA_4_ attenuates myocardial ischemia reperfusion injury via a mechanism related to downregulation of GRP-78 and caspase-12 in rats

**DOI:** 10.1007/s00380-013-0418-y

**Published:** 2013-10-16

**Authors:** Qifeng Zhao, Xingti Hu, Lan Shao, Guowei Wu, Jie Du, Jie Xia

**Affiliations:** 1 Department of Cardiovascular and Thoracic Surgery, The 2nd Affiliated Hospital and Yuying Children’s Hospital of Wenzhou Medical University, 109 College Western Road, Wenzhou, 325027 People’s Republic of China; 2 Department of Cardiovascular Medicine, The 2nd Affiliated Hospital and Yuying Children’s Hospital of WenZhou Medical University, 109 College Western Road, Wenzhou, 325027 People’s Republic of China

**Keywords:** Myocardial reperfusion injury, GRP-78, Caspase-12, Lipoxin, Endoplasmic reticulum stress

## Abstract

This study aims to determine the effect of Lipoxin (LX)A_4_ on myocardial ischemia reperfusion injury (MIRI) in rats and the related molecular mechanisms. Male SD rats were divided into six groups. The sham operation groups (groups C1, C2) were injected with 2 ml/kg normal saline before and after coronary artery threading, respectively. The MIRI group (groups I/R1, I/R2) were injected with normal saline before and after MIRI, respectively. The LXA_4_ groups (groups LX1, LX2) were injected with LXA_4_ before and after MIRI treatment, respectively. The hematoxylin–eosin staining and ultrastructural changes of cardiac muscle were observed. The serum levels of interleukin (IL)-1β, IL-6, IL-10, tumor necrosis factor (TNF) α and cardiac troponin I (cTnI) were measured before open-chest operation and at the end of the experiment. The mRNA and protein levels of GRP-78 and caspase-12 were determined in each group. The myocardial cell apoptosis, myeloperoxidase (MPO), superoxide dismutase (SOD), and malondialdehyde (MDA) contents were detected. The mRNA and protein levels of GRP-78 and caspase-12, the apoptosis, the serum IL-1β, IL-6, IL-10, TNF-α, and cTnI concentrations, MPO, SOD, MDA contents were significantly increased in groups I/R1, I/R2, LX1, and LX2 compared with those in groups C1 and C2 (*P* < 0.05). The mRNA and protein expression levels of GRP-78 and caspase-12 in groups LX1 and LX2 were lower than those in groups I/R1 and I/R2. Compared with group I/R1 and I/R2, the myocardial neutrophil infiltration and ultrastructure damage were significantly less in groups LX1 and LX2. GRP-78 and IL-10 are expressed both extracellularly and intracellularly, but are mainly expressed in the cytoplasms. In the absence of MIRI, LXA_4_ has no detectable effect on GRP-78 and caspase-12 expression. Before and after MIRI, application of LXA_4_ significantly inhibits neutrophil activation, and attenuates myocardial inflammatory injury and oxidative stress. LXA_4_ downregulates the mRNA and protein expression of GRP-78 and caspase-12. LXA_4_ could play a role in myocardial protection via a mechanism related to downregulation of GRP-78 and caspase-12, and inhibition of apoptosis.

## Introduction

Myocardial ischemia/reperfusion injury (MIRI) often occurs in the processes of extracorporeal circulation, thrombolysis, coronary artery bypass grafting, and heart transplantation. MIRI may seriously influence the therapeutic effects and prognosis of cardiac patients by aggravating myocardial injury and causing arrhythmia. The process of MIRI includes alterations in levels of cytokines, neutrophil activation and infiltration, oxygen free radicals, calcium overload, myocardial energy metabolic dysfunction, vascular endothelial cells, and apoptosis [1]. Oxidative stress, calcium overload, and inflammation induce endoplasmic reticulum stress (ERS), while the excessive ERS-mediated myocardial dysfunction and apoptosis further exacerbate MIRI [2–4].

Lipoxins (LXs) are arachidonic acid metabolites formed during inflammation via transcellular biosynthetic pathways. LXs are the first class of lipid mediators that are “switched on” in the resolution phase of an inflammatory response and function as “braking signals” in inflammation. LXs, with dual functions of anti-inflammation and pro-resolution [5], have obvious protective effects on ischemia/reperfusion (I/R) injury of lung, stomach, brain, and kidney [6–9].

It was recently found that atorvastatin and losartan have cardiovascular protective effects on rat heart and aorta [10]. Erythropoietin has recently been shown to confer cardioprotective effects via angiogenesis and antiapoptosis in porcine myocardial injury [11]. Sulfur dioxide can decrease the activated ERS in rats with myocardial injury [12]. Aminobenzoic acid hydrazide, a myeloperoxidase inhibitor, may have protective functions by reducing neutrophil adhesion [13]. In the present study, we tested a hypothesis that administration of LXA_4_ pre-MIRI or post-MIRI attenuates MIRI through the ERS apoptotic pathway in rats.

## Materials and methods

### Rat model of myocardial ischemia–reperfusion injury

The animal experimental procedures were approved by Wenzhou Medical University Animal Care and Use Committee, which is certified by the Chinese Association of Accreditation of Laboratory Animal Care (SYXK, Zhejiang 2010-0150). Sprague–Dawley (SD) male rats (8 weeks old, 200–250 g) were fed a standard diet and maintained in the controlled environment of the animal center at 25 ± 1 °C under a 12 h light–dark cycle, and were used in this study.

In brief, rats were anesthetized by an intraperitoneal injection of 10 % chloral hydrate (300 mg/kg body weight) and placed in a supine position. Blood was collected by femoral venipuncture. Next, the animals were intubated for artificial ventilation with 100 % oxygen using a small animal breathing machine (Tidal volume 5 ml, frequency 70/min) and electrocardiogram (ECG) monitor. Thoracotomy was performed between the sternum and left costa, then the pericardium was gently opened. Myocardial ischemia was induced by ligating the left anterior descending coronary artery (LAD) using a 3-0 silk suture with a section of polyethylene tubing placed over the LAD and 1 mm from the tip of the normally positioned left atrium. The coronary artery was occluded by pulling on the suture tightly 10 min later. After 30 min of myocardial ischemia, reperfusion started by releasing the ligature and removing the tube for 120 min. The chest wall was closed, the animal was extubated, and the body temperature was maintained using a 37 °C warming plate.

The indications of successful LAD occlusion included ST-segment elevation of 0.1 mV or sharp rise in T wave, wider and higher QRS wave of the ECG, and visual cyanosis of myocardial discoloration. The indications of successful reperfusion were ST-segment depression (≥1/2) of the ECG and the myocardial color being normal (pink).

### Animal grouping and treatments

Healthy adult male SD rats were randomly divided into six groups as follows. (1) Using LXA_4_ before I/R (LX1 group): LXA_4_ (100 μg/kg) was injected by femoral vein before thoracotomy. After 30 min of myocardial ischemia, reperfusion was performed by releasing the ligature and removing the tube for 120 min. (2) Using LXA_4_ after I/R (LX2 group): After 30 min of myocardial ischemia, reperfusion lasted for 30 min, then LXA_4_ (100 μg/kg) was injected by femoral vein. Thereafter, reperfusion was initiated again for 90 min. (3) MIRI group 1 (I/R1 group): Normal saline (2 ml/kg) was injected by femoral vein before thoracotomy, with the remaining treatment the same as for the LX1 group. (4) MIRI group 2 (I/R2 group): Normal saline (2 ml/kg) was injected by femoral vein after I/R, with the remaining treatment the same as for the LX2 group. (5) Sham group 1 (C1 group): Rats underwent a similar operation without myocardial I/R, with the remaining treatment the same as for the I/R1 group. (6) Sham group 2 (C2 group): Rats underwent a similar operation without myocardial I/R, with the remaining treatment the same as for the I/R2 group.

In the non-MIRI experiments, the rats were randomly divided into six groups (Sham1, Sham2, Sham3, Sham4, Sham5, and Sham6). In the Sham1 group, the rats were kept for 150 min and then anesthetized, followed by bloodletting. In the Sham2 group, the rats were treated as the same as in the Sham1 group, except that the time before anesthesia was 90 min. In the Sham3 group, rats were injected with 2 ml/kg normal saline. After 150 min, the rats were anesthetized and bloodletting was performed. In the Sham4 group, the rats were treated the same as in Sham3 group, except that the time before anesthesia was 90 min. In the Sham5 group, rats were injected with 100 μg/kg LXA_4_ in 2 ml/kg normal saline. After 150 min, the rats were anesthetized and bloodletting was performed. In the Sham6 group, the rats were treated the same as in the Sham5 group, except that the time before anesthesia was 90 min. The myocardial tissues were collected. The 150- and 90-min intervals before anesthesia mimicked the pretreatment and postprocessing times in the above MIRI experiments.

### Blood collection and tissue harvest

Blood samples were collected in each group immediately before thoracotomy and after anesthetization (*T*_1_) or after the experiments (*T*_2_). In groups C1 and C2, *T*_2_ was obtained after 150 min of placing surgical suture under LAD. For all the other groups, *T*_2_ blood samples were obtained after 120 min of reperfusion. The heart was removed after obtaining blood samples (*T*_2_) and a portion of myocardial tissue was fixed in 4 % formalin. Pathologic examinations of paraffin-embedded sections were performed. A separate portion of myocardial tissue was frozen in liquid nitrogen and kept in the freezer at −70 °C.

### Myocardial tissue hematoxylin–eosin staining

Myocardial tissue slices were baked at a high temperature of 55–65 °C for 1–2 h, followed by xylene dewaxing, alcohol rinsing, and hematoxylin staining for 5 min. The slices were then treated with hydrochloric acid alcohol, added with the bluing agent (Blue Season Sci & Tech Development, Shanghai, China) to promote bluing of the sample, followed by eosin staining for 20 s. Finally, the slices were mounted with neutral gum after dehydration with alcohol and treatment with xylene.

### Transmission electron microscopy (TEM)

For TEM examination, samples containing a 2-mm portion from the edge of the incision were immediately fixed for 4 h in 0.1 M phosphate buffer containing 2.5 % glutaraldehyde and 2 % paraformaldehyde. The samples were then fixed with 1 % osmium tetroxide for 2 h, dehydrated with ethanol, and embedded in epoxy resin. Resin-embedded blocks were cut into 60–80-nm ultrathin sections with an ultra-microtome (PT-XL, RMC Boeckeler, Tucson, AZ, USA). The ultrathin sections were placed on carbon-coated nickel grids and examined with an H-7500 transmission electron microscope (Hitachi, Tokyo, Japan) operated at 80 kV.

### TUNEL assay

Apoptosis was determined by TUNEL assay according to manufacturer’s instructions. Cells with apoptotic morphologic features as well as with tan or brown nuclei were judged to be apoptotic cells. The five fields of view were automatically selected by the Image-Pro Plus version 5.1 image analysis software. The percentage of apoptosis-positive cells was calculated for each field of view. The mean was calculated to obtain the percentage of apoptotic cells, and expressed as apoptotic index. Apoptosis index (%) = (apoptotic nuclei count/total nucleus count) × 100 %.

### Cytokine and cardiac troponin I (cTnI) levels

For cytokine immunoassay, blood samples were collected by femoral venipuncture at the indicated time points before thoracotomy (*T*_1_) and after reperfusion (*T*_2_). The serum levels of interleukin (IL)-1β, IL-6, IL-10, tumor necrosis factor (TNF)-α, and cTnI were measured using a rat enzyme-linked immunosorbent assay (ELISA) kit (Shanghai Boyun Biotech, China) in accordance with the manufacturer’s instructions. Cytokine and cTnI levels were expressed as ng/l.

### Myeloperoxidase (MPO) and superoxide dismutase (SOD) activity

The myocardial MPO activity was determined on frozen tissue by use of colorimetry assay kits (Nanjing Jiancheng Bioengineering Institute, China), and expressed as units per gram tissue wet weight. The myocardial SOD activity was determined on frozen tissue using Xanthine Oxidase assay kits (Nanjing Jiancheng Bioengineering Institute, China), and expressed as units per milligram protein.

### Malondialdehyde (MDA) determination

The MDA content was determined on frozen myocardial tissue by use of the thiobarbituric acid assay kit (Nanjing Jiancheng Bioengineering Institute). The level of MDA was expressed as nanomoles per milligram protein.

### Real-time quantitative polymerase chain reaction (RT-qPCR) analysis

Total RNAs of the tissues were extracted using Trizol Reagent (Invitrogen, Carlsbad, CA, USA) according to the manufacturer’s instructions. Total RNA concentrations were quantified using a spectrophotometer (UV-2000; UNICO, Shanghai, China). Subsequently, 1 ng of total RNA was reverse-transcribed via the cDNA synthesis kit (Invitrogen). RT-PCR was performed using the SYBR green system (Bio-Rad, Hercules, CA, USA). Amplifications for cDNA samples were carried out using a PCR machine, Roto-Gene 3000 (Corbett Robotics, Brisbane, Australia). The primers used in this study are given in Table 1. The relative quantification of target gene was normalized to glyceraldehyd-3-phosphate dehydrogenase (GAPDH), and calculated using the absolute quantification standard curve method. The melting curves were produced at the end of each PCR to confirm the specific amplification. Each sample was analyzed in triplicate.

**Table 1:** Real-time PCR primer sequences

Gene	Forward primer	Reverse primer	Size (bp)
GRP-78	5′-CCTGTTGCTGGACTCTGTGA-3′	5′-GAATACACCGACGCAGGAAT-3′	204
Caspase-12	5′-GCTGCCAAGAGAACACATGA-3′	5′-GGTTCTCAGCTTTGCTCAGG-3′	169
GAPDH	5′-GAGTCAACGGATTTGGTCGT-3′	5′-TTGATTTTGGAGGGATCTCG-3′	238

### Western blot analysis

Equal amounts of protein (50 μg) were subjected to sodium dodecyl sulfate–polyacrylamide gel electrophoresis. Proteins were then transferred to polyvinylidene fluoride membrane. Membrane was blocked with 5 % nonfat milk in Tris-buffered saline, 0.1 % Tween 20 (Sigma, St Louis, MO, USA). The immunoblotting was performed using rabbit antirat GRP-78 (1:500; Cell Signaling Technology, Danvers, MA, USA) and caspase-12 (1:1000; Cell Signaling Technology) as described by the manufacturer. Anti-β-actin was analyzed with the antibody (1:200; Santa Cruz Biotechnology, Santa Cruz, CA, USA). Blots were then developed by incubation with biotinylated antirabbit antibody (1:2000; Vector Laboratories, Burlingame, CA, USA), followed by incubation with the ABC reagent (GE, Fairfield, CT, USA). Signal was detected using an ECL luminescence kit (GE) and X-ray film.

### Immunofluorescence

Rat cardiac tissues were collected and cut into 5-μm slices for immunofluorescence examination. The samples were rinsed with phosphate buffer solution (PBS) for three times and fixed with 4 % (w/v) paraformaldehyde at 4 °C for 2 h. Samples were then permeabilized with dimethylbenzene and blocked with goat serum for 1 h at 37 °C, followed by incubation at 4 °C overnight in a primary antibody solution containing rabbit anti-IL-10 or rabbit anti-GRP-78 BiP antibody (1:70; Cell Signaling Technology). Samples were rinsed with PBS, then incubated with goat anti-rabbit fluorescein isothiocyanate-biotinylated immunoglobulin G secondary antibody (Blue Season Sci & Tech Development). Samples were then observed with a fluorescence microscope (IX71; Olympus, Tokyo, Japan) equipped with ISCapture software, and images were taken with a CCD camera (Discovery C15; Olympus, Tokyo, Japan). For each sample, 10 random microscopic fields were captured and integral optical density (IOD) was calculated.

### Statistical analysis

All data were expressed as mean ± standard deviation. Significant differences were evaluated by one-way analysis of variance to compare more than two groups, or with paired *t* test to compare two groups. The significance level was set at *P* < 0.05. All statistical analyses were performed using SPSS version 17.0 (SPSS, Chicago, IL, USA).

## Results

### Effect of LXA_4_ treatments on the pathologic changes of rat hearts

**Fig. 1:**
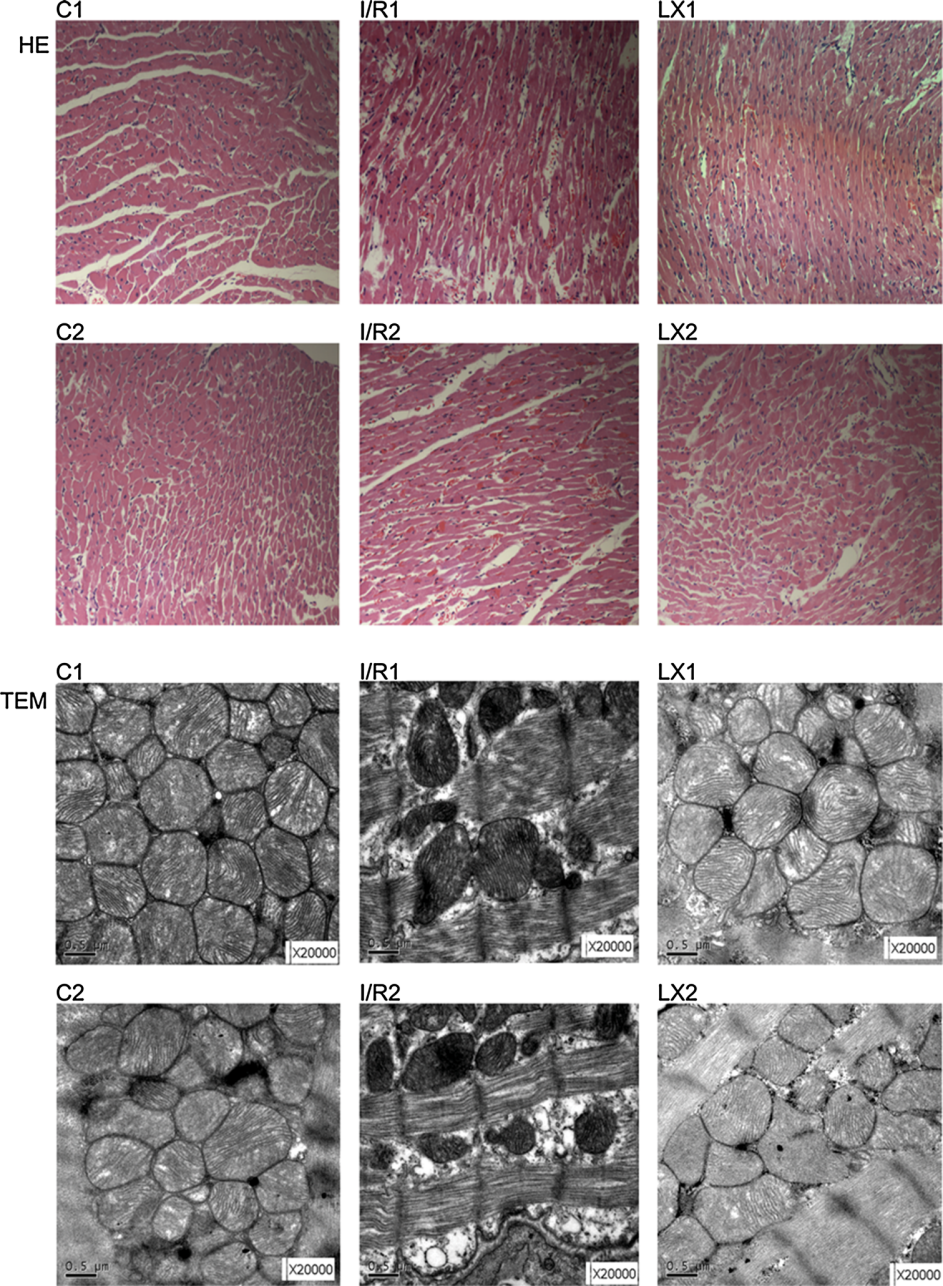
Histologic study (hematoxylin–eosin (HE), ×200) and transmission electron microscopy (TEM) of cardiac tissues from rats in different groups at time point *T*_2_. HE-stain pictures are shown in the upper two panels. In groups C1 and C2, normal-sized cardiomyocytes were observed and no hemorrhage or neutrophil granulocyte infiltration was found. In groups I/R1 and I/R2, cardiomyocyte degeneration, hemorrhage, edema, and significant interstitial neutrophil granulocyte infiltration was observed. In groups LX1 and LX2, there was no significant cardiomyocyte degeneration and no obvious bleeding. Slight edema between the myocardial fibers and mild neutrophil granulocyte infiltration were found. TEM pictures are shown in the lower two panels. In groups C1 and C2, regular myocardial myofilament arrangement, clear intercalated disk structures, normal mitochondrial morphology, and structures with endoplasmic reticulum embedded in myofilament matrix were observed. In groups I/R1 and I/R2, disordered arrangement of myofilaments and a large number of myofilament fractures were observed. The intercalated disk structure was unclear. Abnormal mitochondrial morphology was observed including a high degree of swelling, membrane lysis, fuzzy ridge structure, formation of vacuoles, and endoplasmic reticulum vacuolization. In groups LX1 and LX2, myofilament arrangement was sparse. The intercalated disk structure was slightly fuzzy. Slightly swollen mitochondria were observed

To determine the effect of LXA_4_ on I/R injury of the rat heart, we analyzed the pathologic changes (Fig. 1, the upper panels). In the sham groups (group C1 and group C2), myocardial fibers were regularly arranged with clear striations. No apomorphosis, necrosis, neutrophil infiltration, or other pathologic changes were found. In the nontreated I/R groups (I/R1 and I/R2), local swelling, myocardial necrosis, disorganized myocardial fibers, and ruptured cells were detectible, and a large number of inflammatory cells also appeared in the cytoplasm. In the LXA_4_-treated I/R groups (LX1 and LX2), myocardial cells with normal structure and shape were visible, although the myocardial fibers had mildly swelled and partially ruptured. In addition, slight edema could be observed in the interstitial tissues, with a small amount of inflammatory cells.

### TEM examination of myocardial tissues

TEM images of ultrathin sections of tissue from the rats are shown in Fig. 1 (lower panels). The myocardial cells were detectable in the well-arranged myofilament, and the intercalated disc appeared in the sham groups (groups C1 and C2). Moreover, abundant normal mitochondria with no swelling, normal matrix density, and intact cristae were observed. The round endoplasmic reticulum (ER) can also be observed between the myofilament and the cytoplasm. In groups I/R1 and I/R2, the myocardial I/R induced remarkable ultrastructural damage, which was related to the irregular and edematous separation of myofilament, hypercontraction, and shortening of sarcomeres. Large areas of cytoplasmic vacuolization and mitochondrial swelling also appeared, with decreased matrix density and distortion of cristae. In groups LX1 and LX2, treatment with LXA_4_ showed evident protection with relatively parallel arrangement of myofilaments and normal sarcomeres. Mitochondria were normal with very mild swelling, and matrix density was normal, but there were slightly damaged cristae. However, mild cytoplasmic rarefaction with mild edema could still be observed.

### Inhibitory effect of LXA_4_ on MIRI-induced myocardial cell apoptosis

**Fig. 2:**
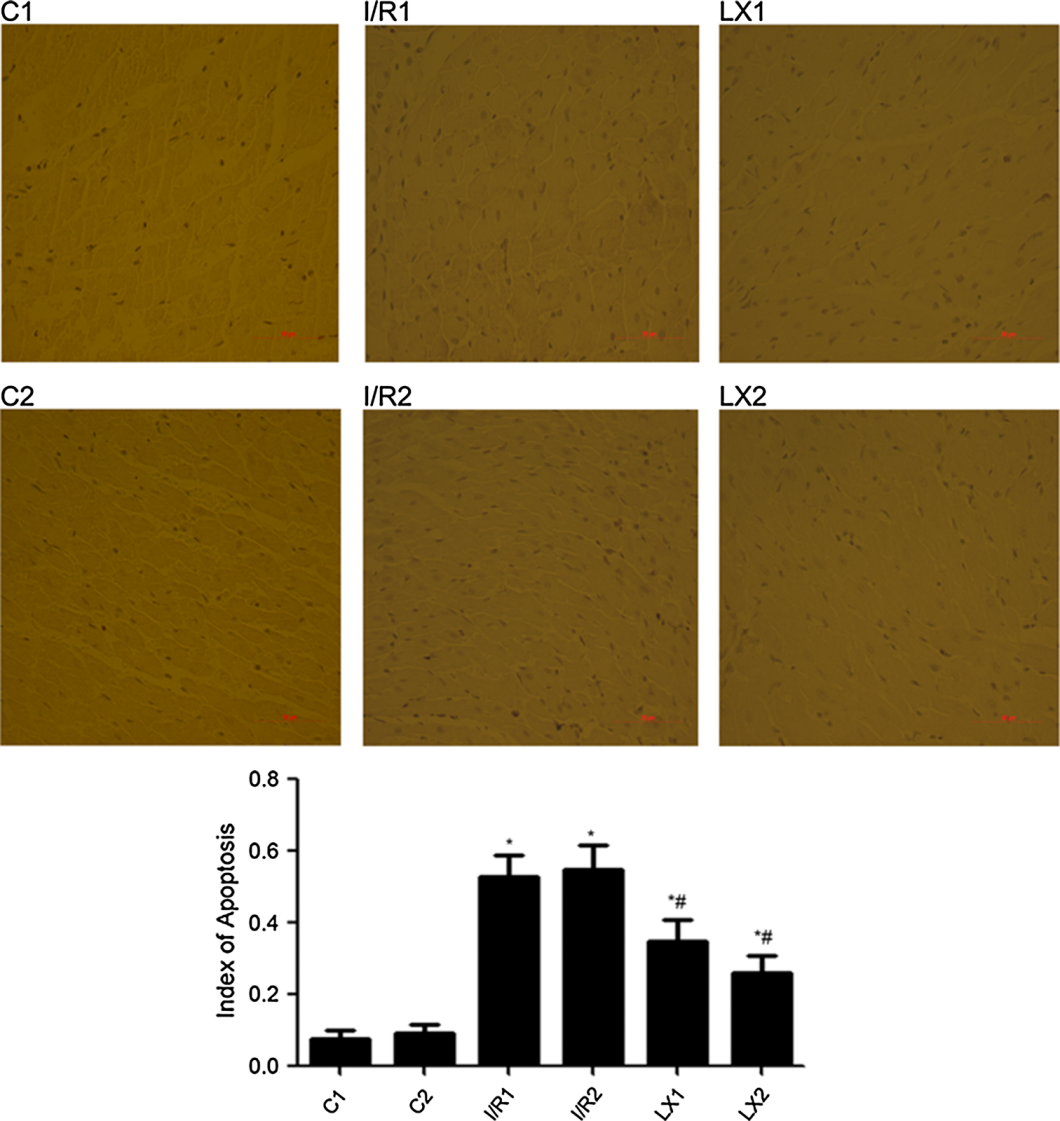
Detection of myocardial cell apoptosis and in cardiac tissues from rats of different groups at *T*_2_. Apoptosis was determined by TUNEL assay according to manufacturer’s instructions. Cells with apoptotic morphologic features and with tan or brown nuclei were judged to be apoptotic cells. Groups C1 and C2: a small amount of apoptotic cells in myocardial tissue; groups I/R1 and I/R2: apoptotic cells in cardiac tissue increased significantly; groups LX1 and LX2: apoptotic cells were between group IR and group LX. Apoptosis index is represented by a histogram. ^*^ *P* < 0.05, comparisons of I/R1 and LX1 groups with C1 group; *P* < 0.05, comparisons of I/R2 and LX2 with C2 group; ^#^ *P* < 0.05, comparisons of LX1 group with I/R1 group, *P* < 0.05, comparisons of LX2 group with I/R2 group

The effect of LXA_4_ on myocardial cell apoptosis was determined. As shown in Fig. 2, in groups C1 and C2 a small amount of apoptotic cells in myocardial tissue was detected. In groups I/R1 and I/R2, the index of apoptotic cells in cardiac tissues were increased significantly (*P* < 0.05) when compared with groups C1 and C2. In groups LX1 and LX2, the index of apoptotic cells was lower than that in groups I/R1 and I/R2 (*P* < 0.05), but higher than that in groups C1 and C2. The results suggest that LXA_4_ inhibits the apoptosis induced by MIRI.

### Effect of different treatments on serum levels of IL-1β, IL-6, IL-10, TNF-α, and cTnI

**Fig. 3:**
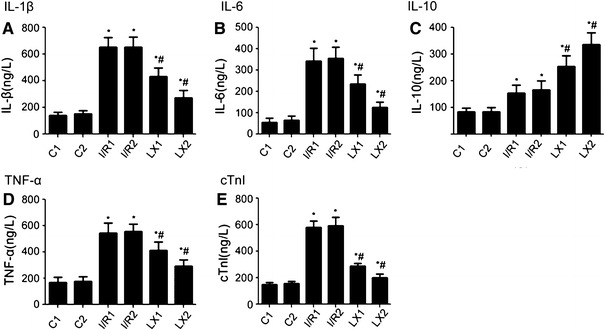
Comparison of concentrations of serum interleukin (IL)-1β, IL-6, tumor necrosis factor-α (TNF-α), IL-10, and cardiac troponin I (cTnI) at *T*_2_ time points among all groups. At time point *T*_2_, blood was collected immediately after the ischemia/reperfusion (I/R) procedure was completed. IL-1β, IL-6, TNF-α, IL-10, and cTnI were measured as described in Materials and methods. **A** IL-1β; **B** IL-6; **C** IL-10; **D** TNF-α; **E** cTnI. ^*^ *P* < 0.05 for comparisons of I/R1 and LX1 groups with C1 group, *P* < 0.05 for comparisons of I/R2 and LX2 with C2 group; ^#^ *P* < 0.05 for comparisons of LX1 group with I/R1 group, *P* < 0.05 for comparisons of LX2 group with I/R2 group

In these experiments, the serum levels of IL-1β, IL-6, IL-10, TNF-α, and cTnI were determined using ELISA kits. It was observed that there was no statistical difference between the groups before thoracotomy. As shown in Fig. 3, after reperfusion the levels of all the cytokines in the I/R1 and LX1 groups were significantly elevated when compared with those of group C1 (*P* < 0.05). Similar results were obtained when the I/R2 and LX2 groups were compared with group C2 (*P* < 0.05). Meanwhile, reduced levels of IL-1β, IL-6, TNF-α, and cTnI, and an increased level of IL-10 in the LX1 and LX2 groups were observed on comparison with I/R1 and I/R2 groups, respectively (*P* < 0.05). The variety of serum IL-1β, IL-6, IL-10, TNF-α, and cTnI concentrations in each group after reperfusion is illustrated in Fig. 3.

### Effects of LXA_4_ on MPO, SOD activity, and MDA production in myocardial tissues

**Fig. 4:**
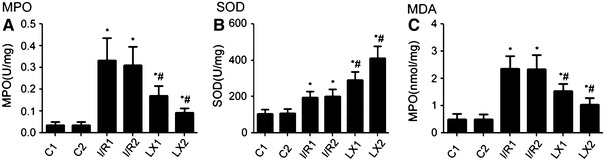
Comparison of cardiac myeloperoxidase (MPO), superoxide dismutase (SOD) activity, and malondialdehyde (MDA) content at *T*_2_ among all groups. At time point *T*_2_, myocardial tissue was collected immediately after the I/R procedure was completed and was kept frozen in liquid nitrogen. MPO, SOD activity, and MDA content were measured as described in Materials and methods. **A** MPO activity; **B** SOD activity; **C** MDA content. ^*^ *P* < 0.05 for comparisons of I/R1 and LX1 groups with C1 group, *P* < 0.05 for comparisons of I/R2 and LX2 groups with C2 group; ^#^ *P* < 0.05 for comparisons of LX1 group with I/R1 group, *P* < 0.05 for comparisons of LX2 group with I/R2 group

The production of MDA, SOD activity, and MPO in myocardial tissues in response to I/R injury was also determined. As shown in Fig. 4, compared with the C1 group, the MDA production, MPO, and SOD activity were increased in the I/R1 and LX1 groups. Similar results were obtained when the I/R2 and LX2 groups were compared with the C2 group (*P* < 0.05). Furthermore, decreased MDA production, decreased MPO, and increased SOD activity in LX1 and LX2 groups was observed in comparison with the I/R1 and I/R2 groups, respectively. Similar results were obtained when the LX2 group was compared with the LX1 group (*P* < 0.05).

### Effects of I/R and LXA_4_ treatments on GRP-78 and caspase-12 gene expression

**Fig. 5:**
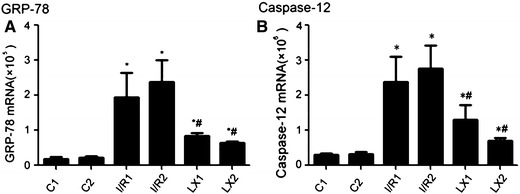
mRNA levels of GRP-78 and caspase-12 of cardiac tissues from rats of different groups at *T*_2_ time points. Samples were collected immediately after the I/R procedure was completed, and real-time quantitative polymerase chain reaction was performed as described in Materials and methods. **A **GRP-78; **B **caspase-12.^*^ *P* < 0.05 for comparisons of I/R1 and LX1 groups with C1 group, *P* < 0.05 for comparisons of I/R2 and LX2 groups with C2 group; ^#^ *P* < 0.05 for comparisons of LX1 group with I/R1 group, *P* < 0.05 for comparisons of LX2 group with I/R2 group

In this experiment, the GRP-78 and caspase-12 gene expression was examined to determine if I/R injury influences the mRNA levels of GRP-78 and caspase-12. As shown in Fig. 5, the real-time RT-PCR results indicated that, compared with the sham groups (C1 and C2 groups), the mRNA level of GRP-78 and caspase-12 in the I/R1, LX1, I/R2, and LX2 groups was increased (*P* < 0.05). The GRP-78 and caspase-12 expression in the LXA_4_-treated LX_1_ and LX_2_ groups was decreased in comparison with the I/R1 and I/R2 groups, respectively (*P* < 0.05). These results suggest that LXA_4_ inhibits the mRNA levels of GRP-78 and caspase-12 in the LXA_4_-treated LX1 and LX2 groups.

### Western blotting analysis of GRP-78 and caspase-12 protein expression

**Fig. 6:**
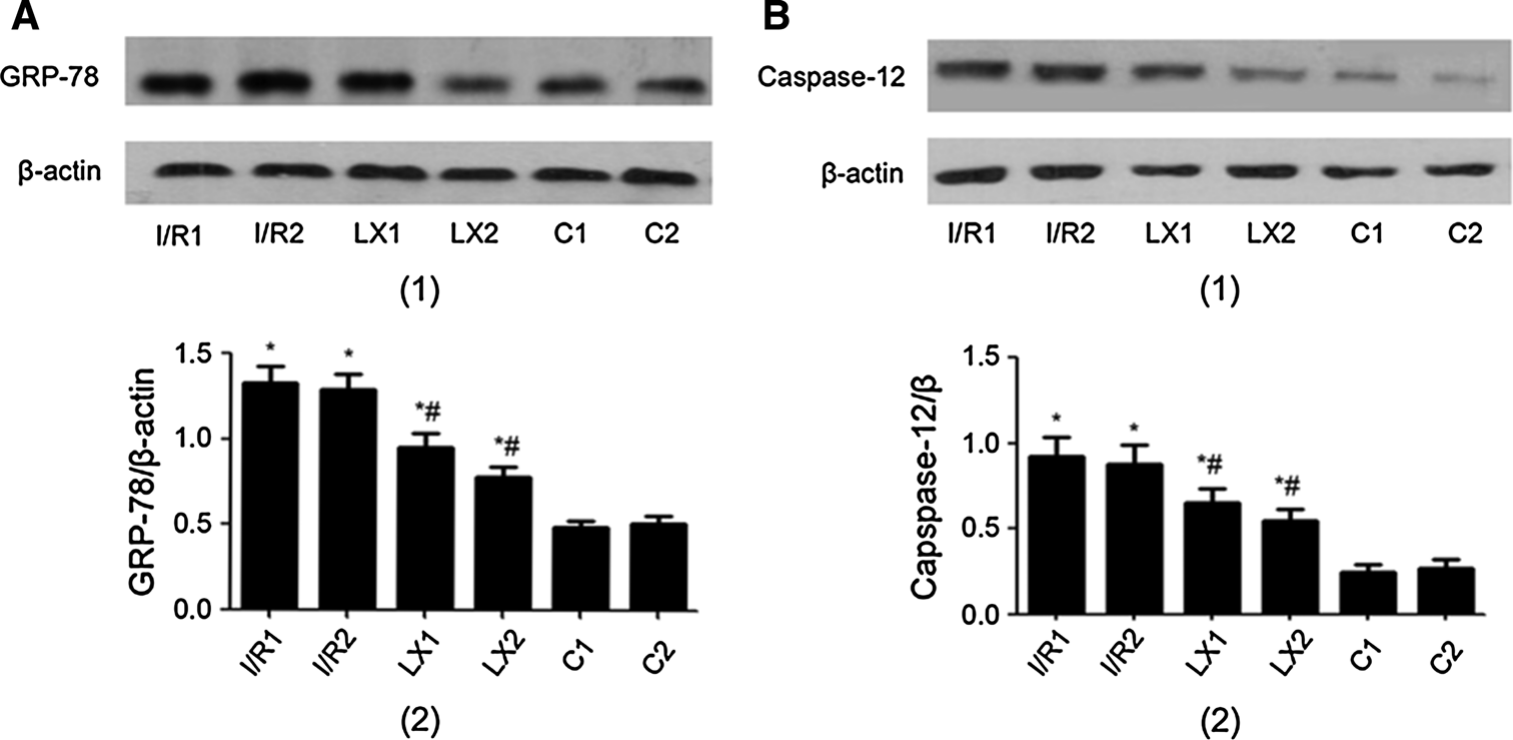
Protein levels of GRP-78 and caspase-12 of cardiac tissues from rats of different groups at *T*_2_ time points. Samples were collected immediately after I/R procedure was completed, and Western blotting was performed as described in Materials and methods. β-Actin was used as a loading control. The lanes shown are representative blots of two independent experiments. **A** GRP-78 (*1, upper*) Western blotting; (*2, lower*) Quantitative data of Western blot. **B** Caspase-12 (*1, upper*) Western blotting; (*2, lower*) Quantitative data of Western blot. ^*^ *P* < 0.05 for comparisons of I/R1 and LX1 groups with C1 group, *P* < 0.05 for comparisons of I/R2 and LX2 groups with C2 group; ^#^ *P* < 0.05 for comparisons of LX1 group with I/R1 group, *P* < 0.05 for comparisons of LX2 group with I/R2 group

In this experiment, the GRP-78 and caspase-12 protein levels were analyzed by Western blotting. As shown in Fig. 6, compared with the sham C1 group, the expression of GRP-78 and caspase-12 in the treated I/R1 and LX1 groups was increased (*P* < 0.05). Similar results were obtained when the I/R2 and LX2 groups were compared with the C2 group (*P* < 0.05). The GRP-78 and caspase-12 protein expression in the LXA_4_-treated LX1 and LX2 groups was decreased in comparison with the saline-treated I/R1 and I/R2 groups, respectively (*P* < 0.05). These results suggest that LXA_4_ inhibits the protein levels of GRP-78 and caspase-12 in the LXA_4_-treated LX1 and LX2 groups.

### Cellular localization of GRP-78 and IL-10 proteins in rat cardiac tissues

**Fig. 7:**
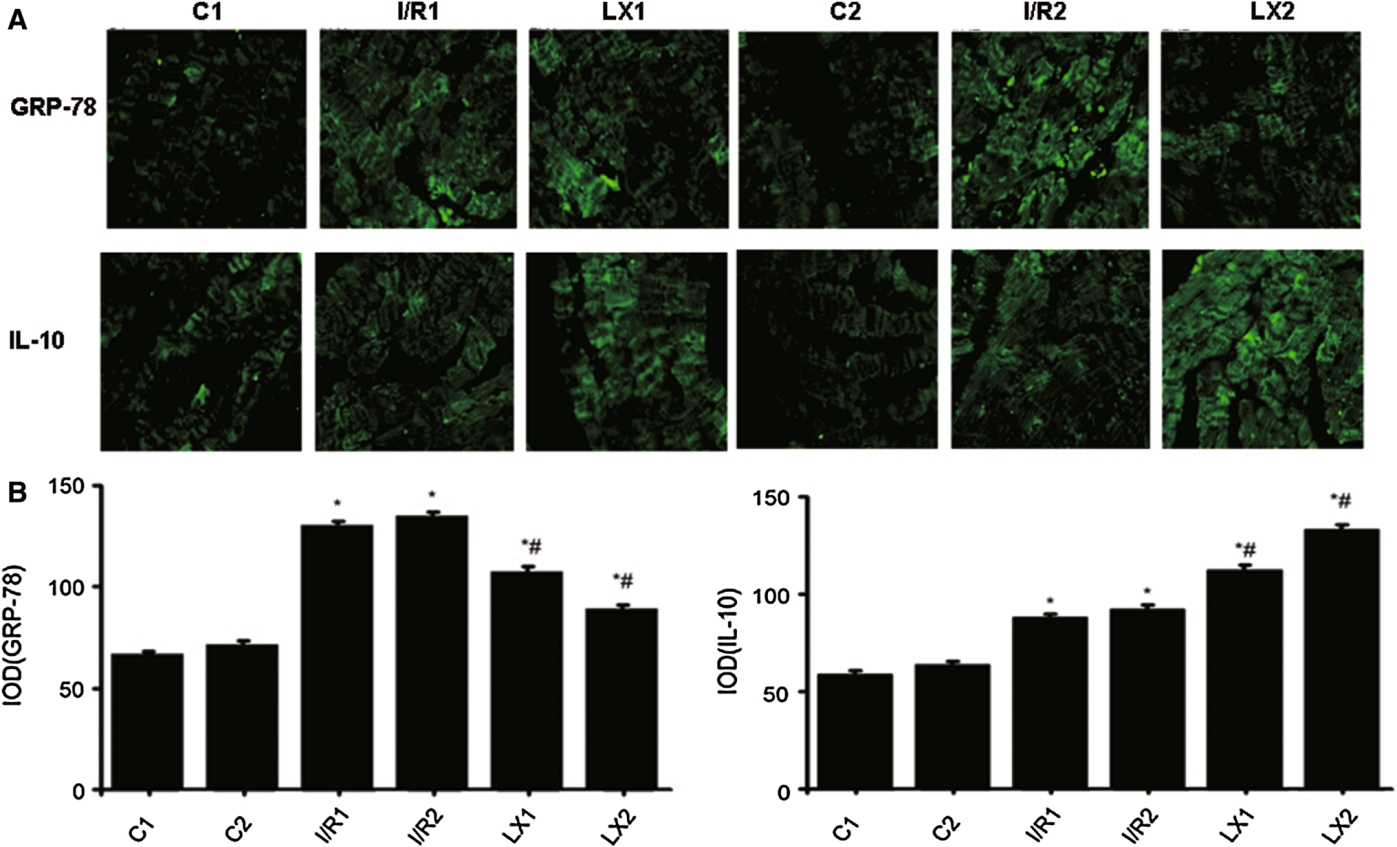
Cellular localization of GRP-78 and IL-10 of cardiac tissues from rats of different groups at *T*_2_ time points. Samples were collected immediately after the I/R procedure was completed, and immunofluorescence detection (×400) was performed as described in Materials and methods. **A** Cellular localization of GRP-78 and IL-10. **B** Integral optical density (IOD) of GRP-78 and IL-10. ^*^ *P* < 0.05 for comparisons of I/R1 and LX1 groups with C1 group, *P* < 0.05 for comparisons of I/R2 and LX2 groups with C2 group; ^#^ *P* < 0.05 for comparisons of LX1 group with I/R1 group, *P* < 0.05 for comparisons of LX2 group with I/R2 group

In these experiments, rat cardiac tissues were collected for immunofluorescence examination. As shown in Fig. 7, the stronger green fluorescence indicated expression of GRP-78 or IL-10 proteins, although weak green fluorescence background was also visible. It was shown that the strong green fluorescence was mainly in muscle filaments. No strong green fluorescence was found within nuclear structures. A few strong green fluorescence dots were visible in the filament-filament connections. These results suggest that GRP-78 and IL-10 are expressed both extracellularly and intracellularly, but mainly in the cytoplasms.

As shown in Fig. 7, the GRP-78 and IL-10 expression levels in I/R1 and LX1 groups were significantly higher than those in the group C1 (*P* < 0.05). GRP-78 and IL-10 expression levels in I/R2 and LX2 groups were also significantly higher than those in the C2 group (*P* < 0.05). The GRP-78 expression levels in the LX1 and LX2 groups were significantly decreased in comparison with those in I/R1 and I/R2 groups (*P* < 0.05). However, the IL-10 expression levels in the LX1 and LX2 groups were significantly increased in comparison with those in I/R1 and I/R2 groups (*P* < 0.05). These results suggest that LXA_4_ inhibits the protein levels of GRP-78 but increases the IL-10 levels.

### In the absence of MIRI, LXA_4_ has no detectable effects on GRP-78 and caspase-12 expression

**Fig. 8:**
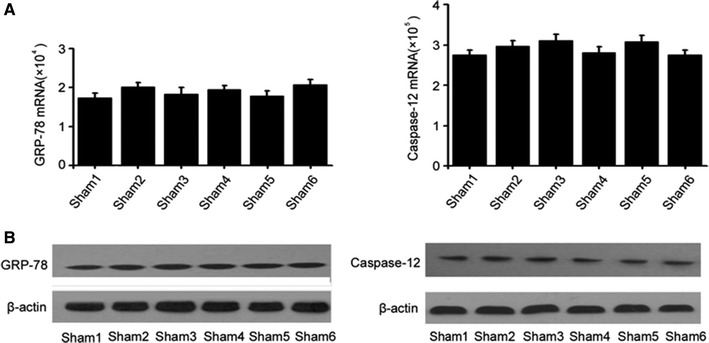
RT-qPCR analyses and Western blotting of myocardial GRP-78 and caspase-12 in rat cardiac tissues from the non-MIRI experiments. In the non-MIRI experiments, the rats were randomly divided into six groups (Sham1, Sham2, Sham3, Sham4, Sham5, and Sham6). The myocardial tissues were collected. The 150- and 90-min intervals before anesthesia mimicked the pretreatment and postprocessing times in these MIRI experiments.It was observed that there was no statistical difference among all groups. **A** Samples were collected and RT-qPCR was performed as described in Materials and methods. **B** Protein levels were detected by Western blotting. β-Actin was used as a loading control

To determine the effect of LXA_4_ in the absence of MIRI, rats without MIRI were used and treated with LXA_4_ as described in Materials and methods. Quantitative real-time PCR and Western blotting were performed to detect the mRNA and protein levels of myocardial GRP-78 and caspase-12 in cardiac tissues from rats of different groups. As shown in Fig. 8, there were no statistically significant differences in the mRNA (Fig. 8A) and protein expression levels (Fig. 8B) of GRP-78 and caspase-12 among the groups. These results suggest that in the absence of MIRI, LXA_4_ has no detectable effects on GRP-78 and caspase-12 expression.

## Discussion

The ER is a highly dynamic organelle that plays a central role in lipid and protein biosynthesis. The ER has multiple cellular functions in the synthesis of integral membrane proteins, proper folding and oligomerization of proteins, and Ca^2+^ storage and signaling. ERS is a subcellular pathologic process of imbalance in ER homeostasis, which can be caused by the disturbances in redox regulation, calcium regulation, and glucose deprivation. ER can be very sensitive to various stresses. Through ERS, cells can be automatically self-protected to a certain extent.

ERS can be induced by MIRI, leading to the imbalance of ER homeostasis. However, different stages of ERS determine divergent cell fates [14]. In the early stage of ERS, the misfolded proteins accumulate and trigger the release of GRP-78 and then launch the unfolded protein response (UPR). UPR is an evolutionarily conserved response that can be activated to enhance the protein-folding capacity of the ER and promote ER-associated protein degradation to remove the misfolded proteins. Therefore, the release of GRP-78 is critical in the early protective response, which is an upstream gene in the ERS signaling and can also be seen as a marker in the early ERS [15]. In our study, the increased mRNA and protein expression of GRP-78 induced by I/R demonstrated the process of ERS, which can protect the ER and further maintain ER homeostasis. Immunofluorescence also confirmed that the expression of GRP-78 in the cytoplasm increased significantly after MIRI. However, persistent existence of excessive stress inhibits the re-establishment of ER homeostasis and triggers cell death, usually via inducing apoptosis. The chaperone GRP-78 will then bind to other proteins. In rodents, caspase-12 separated from GRP-78 complex will be activated and will trigger ERS apoptosis [16, 17]. It was also observed that compared with the control group, caspase-12 expression was upregulated and the incidence of myocardial apoptosis increased, suggesting that MIRI induces myocardial apoptosis through the ERS apoptotic pathways.

Myocardial cell apoptosis is one of characteristics of MIRI damage, and plays a crucial role in MIRI. Several pathways linking ERS to cell death have been reported, although the related mechanisms have not been clearly identified. However, in rodents the activation of ERS apoptotic pathways specific for protein caspase-12 in ER membrane has been observed during apoptosis [18]. Lee et al. found that caspase-12 was particularly activated in ERS but that no activation of caspase-12 was detected in non-ERS-mediated apoptosis [19]. Wang et al. reported that cardiac muscles can be protected by inhibiting the ERS and ERS-related apoptosis [20]. It was shown in this study that the downregulated myocardial cell apoptosis and cTnI expression, as well as the reduced expressions of GRP-78 and caspase-12 after using LXA_4_, indicate that the protective effective of LXA_4_ on cardiac muscle may be related to the ERS. LXA_4_ can inhibit excessive ERS and maintain the function of ER, resulting in reduced expression of GRP-78 and caspase-12. Consequently, myocardial cell apoptosis can be alleviated, which also provides new insight into the prevention and treatment of MIRI.

However, the mechanism underlying the effect of LXA_4_ on ERS is still not clear. Hayashi et al. considered that ER is sensitive to oxidative stress and that brain I/R injury-produced active oxygen may lead to ER damage [21]. Kumar and Sitasawad discovered that ER could be the possible target of active oxygen [22]. LXs are newly discovered bioactive products derived from arachidonic acid that have a number of proinflammatory and anti-inflammatory functions. LXs also inhibit neutrophil granulocyte infiltration and the production of reactive oxygen. LXA_4_ can inhibit the production of NADPH oxidase-mediated reactive oxygen in BV2 cells [23] and the recruitment of neutrophils to the inflammatory sites [24]. The MPO activity in lung tissue after I/R can also be reduced by LXA_4_ [6]. Meanwhile, LXA_4_ can suppress the expression of IL-6, TNF-α, and IL-8, and upregulate the IL-10 expression of vascular endothelial growth factor-stimulated inflammation in human umbilical vein endothelial cells [25]. In vivo study also showed that LXA_4_ can restrain TNF-α-induced I/R injury through upregulation of IL-10 in IL-10(–/–) mice [26].

In this study, with the treatments of LXA_4_, the expression of proinflammatory cytokines (IL-1β, IL-6, IL-8, and TNF-α) were reduced while the anti-inflammatory cytokine IL-10 were upregulated, leading to the balance of pro-inflammatory/anti-inflammatory cytokines and a less inflammatory response. The increased expression of MPO, SOD, and MDA in the I/R groups indicates that peroxidative stress has been induced after I/R, suggesting that the decreased scavenging ability of free radicals may further lead to myocardial injury. After the treatments of rats with LXA_4_, the SOD activity increased significantly and the expression of MPO and MDA was markedly reduced, suggesting that the reduced MIRI may be due to the decreased expression of the lipid peroxidation caused by oxygen free radicals. The attenuated neutrophil infiltration may also play a role in restoring the oxidant/antioxidant balance after I/R injury. LXA_4_ can protect the ultrastructure of ER and mitochondria. It has been reported that LXA_4_ can remarkably inhibit the release of mitochondria-mediated apoptosis proteins and the activation of caspase [27]. Research showed that antioxidants can alleviate ERS injury through eliminating the oxygen free radicals and decreasing the production of GRP-78 and caspase-12 [28]. The proinflammatory cytokines are able to cause cardiac injury through cell apoptosis and may influence the remodeling of the extracellular matrix [29]. Therefore, we assume that the attenuated excessive ERS and cell apoptosis by LXA_4_ may be caused by the restraint of neutrophil activation and further inflammatory injury.

In conclusion, the application of LXA_4_ pre-MIRI and post-MIRI apparently inhibited the activation of neutrophils, alleviated oxidative damage of cardiac muscle, reduced the expression of GRP-78 and caspase-12, prevented the excessive ERS-mediated cell apoptosis, and therefore effectively protected the cardiac muscles. Although both pharmacologic preconditioning and postconditioning with LXA_4_ can protect cardiac muscle from MIRI, the preconditioning has been largely restrained because of the poor prediction of MIRI in clinical terms, implicating that the postcondition treatment of LXA_4_ should have great potential for clinical application in MIRI in the future. Further studies to optimize the dose, timing, and drug administration of LXA_4_ are warranted.
